# Isolation and Characteristics of Shiga Toxin 2f-Producing *Escherichia coli* among Pigeons in Kyushu, Japan

**DOI:** 10.1371/journal.pone.0086076

**Published:** 2014-01-23

**Authors:** Koichi Murakami, Yoshiki Etoh, Sachiko Ichihara, Eriko Maeda, Shigeyuki Takenaka, Kazumi Horikawa, Hiroshi Narimatsu, Kimiko Kawano, Yoshiaki Kawamura, Kenitiro Ito

**Affiliations:** 1 Department of Health Science, Fukuoka Institute of Health and Environmental Sciences, Dazaifu, Fukuoka, Japan; 2 Laboratory of Microbiology, Oita Prefectural Institute of Health and Environment, Oita, Oita, Japan; 3 Miyazaki Prefectural Institute for Public Health and Environment, Miyazaki, Miyazaki, Japan; 4 Department of Microbiology, School of Pharmacy, Aichi Gakuin University, Chikusa-ku, Nagoya, Aichi, Japan; 5 National Institute of Infectious Diseases, Shinjuku-ku, Tokyo, Japan; Beijing Institute of Microbiology and Epidemiology, China

## Abstract

An increasing number of Shiga toxin 2f-producing *Escherichia coli* (STEC2f) infections in humans are being reported in Europe, and pigeons have been suggested as a reservoir for the pathogen. In Japan, there is very little information regarding carriage of STEC2f by pigeons, prompting the need for further investigation. We collected 549 samples of pigeon droppings from 14 locations in Kyushu, Japan, to isolate STEC2f and to investigate characteristics of the isolates. Shiga toxin *stx*_2f_ gene fragments were detected by PCR in 16 (2.9%) of the 549 dropping samples across four of the 14 locations. We obtained 23 STEC2f-isolates from seven of the original samples and from three pigeon dropping samples collected in an additional sampling experiment (from a total of seven locations across both sampling periods). Genotypic and phenotypic characteristics were then examined for selected isolates from each of 10 samples with pulsed-field gel electrophoresis profiles. Eight of the *stx*_2f_ gene fragments sequenced in this study were homologous to others that were identified in Europe. Some isolates also contained virulence-related genes, including *lpfA*_O26_, *irp*_2_, and *fyuA*, and all of the 10 selected isolates maintained the *eae*, *astA*, and *cdt* genes. Moreover, five of the 10 selected isolates contained *sfpA*, a gene that is restricted to Shiga toxin-producing *E. coli* O165:H2 and sorbitol-fermenting Shiga toxin-producing *E. coli* O157:NM. We document serotypes O152:HNM, O128:HNM, and O145:H34 as STEC2f, which agrees with previous studies on pigeons and humans. Interestingly, O119:H21 was newly described as STEC2f. O145:H34, with sequence type 722, was described in a German study in humans and was also isolated in the current study. These results revealed that Japanese zoonotic STEC2f strains harboring several virulence-related factors may be of the same clonal complexes as some European strains. These findings provide useful information for public health-related disease management strategies in Japan.

## Introduction

Recent reports have shown that cases of human infection by Shiga toxin 2f-producing *Escherichia coli* (STEC2f) are becoming more frequent, with pigeons being implicated as a possible source of infection [1], [2]. For example, van Duynhoven *et al.* [3] screened 211 human stool specimens suspected to contain Shiga toxin-producing *E. coli* (STEC), and reported that three of seven STEC strains were positive for STEC2f. Prager *et al.* [1] also reported that STEC2f was present in 2.5% of STEC strains identified in their study. In Japan, STEC2f strain O128:HNM was isolated from the feces of a 1-year-old boy with diarrhea and abdominal pain in 2002 [4]. In addition, Sonntag *et al*. [5] showed that a STEC2f O128:HNM strain from human diarrhea was identical to those of pigeon isolates belonging to the serotype O128:H2, based on the restriction fragment length polymorphism pattern identified in Germany. These studies have shown that the occurrence of STEC2f in humans is clearly higher than previously thought, and that zoonotic transfer from pigeons is a potentially serious threat.

In Europe, STEC2f strains among pigeons and humans were investigated using several methods, including isolation of the pathogen from city pigeons, analysis of the isolates' characteristics, and relationships between isolates. Isolation of *stx*_2f_ (or the toxin, Stx2f) from pigeon droppings was reported in Europe [6]. STEC2f serotypes are variable, and so far include the O15, O18ab:HNM, O25:H7, O45, O45:HNM, O66:HNM, O75:HNM, O128:H2, O132, O135:HNM, O152:HNM, and O-untypeable serotypes in Japanese [7] and European pigeons [5], [8], [9]. The *stx*_2f_ sequences were analyzed [2] and multi-locus sequence typing (MLST) of the pathogen in humans was conducted [1]. Given that the capacity of STEC strains to cause human disease is strongly associated with other virulence-related genes, in addition to *stx* [10], Sonntag *et al*. [5] evaluated the potential virulence of STEC2f strains containing such virulence-related genes, including *lpfA*_O26_ (major subunit of long polar fimbriae of STEC O26), *sfpA* (major fimbrial subunit of Sfp fimbriae), *irp*_2_ (iron-repressible protein 2), and *fyuA* (ferric yersiniabactin uptake receptor). Other virulence-related genes, such as *eae* (intimin), EHEC-*hlyA* (enterohemorrhagic *E. coli* hemolysin), and *cdt* (cytolethal distending toxin), were also detected in STEC2f strains in Europe [1]; however, there is only one study of this emerging pathogen in Japan, which sampled only 67 pigeons [7]. The purpose of the present study was to investigate the prevalence of STEC2f-harboring pigeons in Kyushu, Japan, to determine the phenotypic and genotypic characteristics of any resulting isolates, and to compare the genotypes, including virulence-related genes, of Japanese isolates with those reported in Europe. Accordingly, we successfully isolated STEC2f strains from the pigeon droppings and identified some overlap with virulence gene clones identified in Europe, as well as documenting serotype O119:H21 as STEC2f for the first time. This study represents a critical appraisal of the occurrence of pigeons infected with the STEC2f zoonotic pathogen in Japan.

## Materials and Methods

### Sampling of pigeon droppings

**Figure 1:**
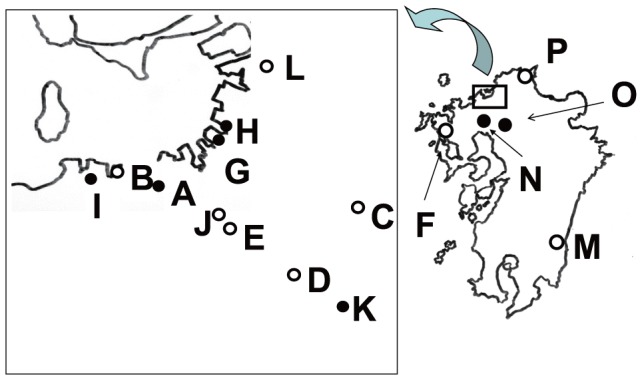
Sampling locations in surveillance experiments in Kyushu, Japan. Closed circles show locations of Shiga toxin 2f-producing *Escherichia coli*. Open circles indicate locations with no Shiga toxin 2f-producing *E. coli*.

Samples of droppings from city pigeons were collected for both experiment 1 (single dropping collection) and experiment 2 (pooled dropping collection) (see below) in Kyushu (Fig. 1), Japan, between December 2008 and March 2009. Kyushu is Japan's third largest island. It is located southwest of the main island of Honshu. For both experiments, droppings were collected from the ground while the pigeons were eating feed that we supplied, except at location K, where droppings were collected from plastic boards that had been sterilized with ethyl alcohol (80%). Therefore, not every sample came from a separate bird. Sampling locations were selected randomly from parks, station squares, and religious sites. In experiment 1, 549 individual pigeon droppings were collected from 14 locations in Kyushu (A–E, G–I, K–P in Fig. 1). Each dropping was collected in a 15-mL conical tube. In experiment 2, which was carried out for additional collection of STEC2f-isolates, 19 samples consisting of pooled pigeon droppings were collected from 13 locations (A–L, P in Fig. 1) in Kyushu.

### Detection of *stx*_2f_ gene and isolation of STEC2f from pigeon droppings

Each dropping sample for experiment 1 was mixed with 10 mL of buffered peptone water (Oxoid, Hampshire, UK), while each pooled sample in experiment 2 was mixed with 225 mL of buffered peptone water. The collected samples in the buffered peptone water were incubated overnight at 42°C to reflect the body temperature of pigeons. Aliquots (0.1 mL in experiment 1 and 1 mL in experiment 2) of the cultures were centrifuged at 3,220× *g* for 20 min, and pellets were re-suspended in 0.5 mL of Tris-EDTA buffer. Cell suspensions were then boiled for 10 min and centrifuged at 3,220× *g* for 20 min for experiment 1 and at 20,814× *g* for 10 min for experiment 2. A 1-µL aliquot of each supernatant was used as a template for PCR-based detection of *stx*_2f_. PCR was performed as described previously by Nakao *et al*. [11]. Primers G3-F (5′-TTT ACT GTG GAT TTC TCT TCG C-3′) and G3-R (5′-TCA GTA AGA TCC TGA GGC TTG-3′), at a final concentration of 0.5 µM, and Go*Taq* Green Master Mix (Promega, Madison, WI, USA) were used in the PCR reaction mixture.

The *stx*_2f_ PCR-positive mixed cultures were streaked onto deoxycholate-hydrogen sulfide-lactose agar (Eiken Chemical Co., Tokyo, Japan) and the plates were incubated at 37°C overnight. Presumptive *E. coli* colonies (25–96/sample) were isolated on nutrient agar plates (Eiken Chemical Co.) and incubated at 37°C overnight. The isolates were then re-tested for *stx*_2f_ using the PCR method described above. The biochemical characteristics of the *stx*_2f_-positive isolates were examined using triple sugar iron agar (Eiken Chemical Co.), lysine decarboxylase broth (Eiken Chemical Co.), sulfide indole motility medium agar (Eiken Chemical Co.), methyl red-Voges-Proskauer medium (Nissui Pharmaceutical Co., Tokyo, Japan), Simmons citrate agar (Eiken Chemical Co.), and cytochrome-oxidase test strips (Nissui Pharmaceutical Co.) to determine whether the isolates were *E. coli*. Serological characteristics of the isolates were investigated using O- and H-antisera (Denka Seiken Co., Tokyo, Japan). The isolates were also tested using a previously described PCR-based method for discriminating between *E. coli* and *Escherichia albertii* [12], [13]. Genotyping of flagella antigen (H-typing), from isolates that were identified as having a type other than H- in the phenotyping assay, was also carried out by restriction fragment length polymorphism analysis, as previously described [14].

### Pulsed-field gel electrophoresis

All of the STEC2f isolates (n = 23) were tested with pulsed-field gel electrophoresis (PFGE) to select representative isolates from each sample for further characterization. PFGE was carried out as previously described [15] for 11 isolates, while the other 12 isolates that showed Tris-dependent degradation were subsequently analyzed by PFGE with 50 µM thiourea described byRömling and Tümmler [16].

### Full-length sequencing of *stx*_2f_

Full-length sequence analysis of the *stx*_2f_ genes from the 10 selected isolates was carried out as previously described [17]. The sequences were assembled using the SeqManII program in the Lasergene software package (DNASTAR, Madison, WI, USA), and were compared with *stx*_2f_ reference sequences (AB232172, M29153, AJ010730, AJ270998, and AB472687 from the DNA Data Bank of Japan, or DDBJ). Cluster analysis was performed using the unweighted pair-group method with arithmetic averages using MEGA 4 software [18].

### Genotypic and phenotypic characterization of isolates

The following genes were amplified using primers described by Sonntag *et al*. [5]: *lpfA*_O26_, *iha* (iron-regulated gene A homologue adhesin), *efa1* (enterohemorrhagic *E. coli* factor for adherence), *sfpA*, EHEC-*hlyA*, *espP* (extracellular serine protease), *pagC* (outer membrane invasion protein), *irp*_2_, and *fyuA*. *cdt* was amplified using the method described by Pickett *et al*. [19], and *eae*, *bfpA* (major pilin structural unit bundling), and *astA* (heat-stable toxin 1) were amplified as described by Kobayashi *et al.* [20]. Heteroduplex mobility assay typing of the 5′ terminal region of the *eae* gene was performed as described by Ito *et al*. [21]. Typing of the 3′ terminal region of *eae* was conducted using the PCR method described by Blanco *et al.* [22]. Full-length sequence analysis of the *sfpA* genes from isolates G2-1c, G5-1, H1-1b, H3-1a, and H4-1b was carried out as previously described [17] using novel primers designed for this study, AJ131667-1036F: 5′-CATAAGCACAGAGAGCAGACTAAC-3′ and EU980315-1479R: 5′-GCAGCACTCCAGAGAAATGTTAC-3′ to confirm whether the genes detected by PCR were *sfpA*.

### Toxin assay

Filtrates of cultures of each of the isolates, with mitomycin C (MMC) (0.2 mg/L), were tested for cytotoxic activity in Vero cells [23], and in a reversed passive latex agglutination assay (RPLA) using a VTEC-RPLA kit (Denka Seiken) to detect Shiga toxin, as previously described [17]. *E*. *coli* O157:H7 (ATCC 43894), harboring *stx*_1_ and *stx*_2_, was also examined as a reference strain in both tests.

### Multi-locus sequence typing (MLST)

Housekeeping genes *adk* (adenylate kinase), *fumC* (fumarate hydratase), *gyrB* (DNA gyrase), *icd* (isocitrate/isopropylmalate dehydrogenase), *mdh* (malate dehydrogenase), *purA* (adenylosuccinate dehydrogenase), and *recA* (ATP/GTP binding motif) were used for MLST analysis. Cell treatment and sequencing were performed as previously described [24]. Primers for the sequencing reactions are listed in the MLST Database at the Environmental Research Institute, University College Cork, Ireland (http://mlst.ucc.ie/, available April 1, 2012). Sequences were submitted to the database website and were assigned existing or novel allele type numbers and sequence type (ST) numbers defined by the database. Composite STs were assigned based on the set of allele types derived from each of the seven loci. Allele sequences for each strain were then concatenated in the order *adk*–*fumC*–*gyrB*–*icd*–*mdh*–*purA*–*recA* for a final composite length of 3,423 bp. The concatenated sequences were aligned using ClustalW software [25], and a phylogenetic tree was constructed using the neighbor-joining method to compare with other published STs (Center for Information Biology and DDBJ). Phylogenetic analyses were performed using NJplot (http://pbil.univ-lyon1.fr/software/njplot.html). A minimum spanning tree generated from the data for ST 20, ST 2684, ST 2685, ST 28, ST 722, ST 40, and ST 11 was also described using Prim's algorithm from the PubMLST site (http://pubmlst.org/, accessed May 2, 2013).

### Ethics statement

No specific permits were required for the described field studies, locations, and activities. The sampled locations are not privately-owned or protected in any way, and the field studies did not involve endangered or protected species. At no point did the researchers come into physical contact with the pigeons. This study was carried out in strict accordance with the guidelines of the Regulations for the Ethical and Humane Use of Experimental Animals at Fukuoka Institute of Health and Environmental Sciences, which is based on domestic standards, and approved by the Animal Ethics Committee of Fukuoka Institute of Health and Environmental Sciences under permit number 250802.

## Results

### Detection of Shiga toxin 2f-producing *E. coli*

In experiment 1, *stx*_2f_ was detected in 16 (2.9%) of 549 dropping cultures from four of the 14 locations by PCR. The incidence varied from 0–17.0% (Table 1). In experiment 2, *stx*_2f_ was detected in four of 19 pooled dropping samples at four (A, H, I and K) of the 13 locations. *stx*_2f_ was detected in one out of the two samplings and one out of five samplings at locations I and K, respectively. The combined results of both the experiments showed *stx*_2f_ was detected at seven of the 16 locations (Fig. 1). No common characteristics or features were found in the seven locations where *stx*_2f_ was detected.

**Table 1:** stx_2f_ PCR-positive droppings from pigeons from 12 locations in Kyushu, Japan (experiment 1).

Sampling location	No. of pigeon droppings tested	No. of PCR-positive droppings (%)	
G	47	8	(17.0%)
H	47	4	(8.5%)
N	50	3	(6.0%)
O	25	1	(4.0%)
A	49	0	(0.0%)
B	6	0	(0.0%)
C	50	0	(0.0%)
D	29	0	(0.0%)
E	50	0	(0.0%)
I	50	0	(0.0%)
K	8	0	(0.0%)
L	50	0	(0.0%)
M	38	0	(0.0%)
P	50	0	(0.0%)
Total	549	16	(2.9%)

### Phenotyping and genotyping of selected isolates chosen by pulsed-field gel electrophoresis

We obtained 23 STEC2f isolates from 10 samples at seven locations in total: 14 isolates from seven samples from four locations in experiment 1, and nine isolates from three samples from three locations in experiment 2. These isolates were identified as STEC2f-positive by PCR and as *E. coli* by biochemical tests. None of the isolates were identified as *E. albertii* by PCR [12], [13].

**Figure 2:**
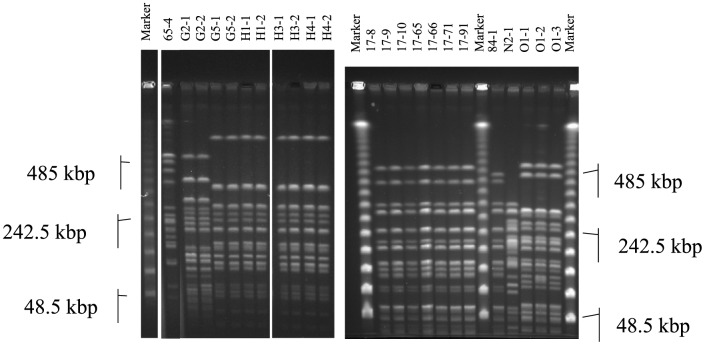
Comparison of pulsed-field gel electrophoresis analysis of fingerprints from Shiga toxin 2f-producing *Escherichia coli* isolates digested with *Xba*I. Isolates 65-4 to H4-2 were tested with Tris-borate-EDTA buffer. Isolates 17-8 to O1-3 were tested using buffer with 50 µM thiourea because they showed degradation in the presence of Tris.

In the PFGE analyses, no remarkable differences in fragment patterns (defined as more than three fragments [26]) were observed between isolates from the same samples, suggesting that these isolates were the same or closely related strains (Fig. 2). Isolates from sample location G (G5 in Fig. 2) and H (H1, H2, and H3 in Fig. 2) showed the same PFGE profiles. Therefore, we selected one isolate from each of the 10 unique samples covering all STEC2f-positive locations (n = 7) across the two experiments. The 10 selected STEC2f-isolates showed several serotypes and characteristics (Table 2). The results of genotyping and phenotyping H-type assays were identical in each of six isolates. The 10 selected isolates showed a variety of O serogroups that were specific to location, and no O serogroup was shared between the different locations, except for untypeable. Selected isolates from each sample showed the following characteristics: supernatants from the isolate cultures containing MMC had titers of 16–256 in the RPLA test for Stx2 only, while ATCC 43894, the control strain, had titers of 16 and 8,192 for Stx1 and Stx2, respectively, in the MMC treatment assay. For the Vero cell assays using culture supernatants, the titers were between 4,096 and 262,144 with MMC, while ATCC 43894 with MMC treatment showed a titer of 65,536 (Table 2). Normal Vero cells were observed in a control plate containing only medium and MMC.

**Table 2:** Characteristics of Shiga toxin 2f-producing *Escherichia coli* isolates that were isolated in this study.

Isolate No.	Location	Serotype	*stx*_2f_ sequence type	Sequence types from multi locus sequence typing	Gene detection*							*eae* typing		Toxin assay (titer)	
					*astA*	*lpfA*_O26_	*sfpA*	*irp*_2_	*fyuA*	*cdt*	EHEC-*hlyA*, *bfpA*, *iha*, *espP*, *efa1*, or *pagC*	Heteroduplex mobility assay typing in 5′ region	3′ region typing	Vero cell assay +MMC 0.2 mg/L†	RPLA +MMC 0.2 mg/L
N2-1	N	O152:HNM	Type A‡	2685	+§	+	−§	−	−	+	−	b1	β 1	262,144	256
O1-3	O	O128:HNM	Type B‡	2684	+	+	−	−	−	+	−	b1	β 1	Not done	Not done
84-1	K	OUT:HNM‖	Type A	20	+	+	−	−	−	+	−	b1	β 1	Not done	Not done
17-8	A	OUT:HNM	Type A	20	+	+	−	−	−	+	−	b1	β 1	16,384	64
G2-1c	G	O119:H21	Type A	40	+	−	+	−	−	+	−	d1	θ/γ 2	262,144	64
65-4	I	O145:H34	Type B	722	+	−	−	−	−	+	−	a1	ι1	4,096	16
H1-1b	H	OUT:H6	Type A	28	+	−	+	+	+	+	−	a1	ξ/β 2	262,144	256
H3-1a	H	OUT:H6	Type A	28	+	−	+	+	+	+	−	a1	ξ/β 2	262,144	256
H4-1b	H	OUT:H6	Type A	28	+	−	+	+	+	+	−	a1	ξ/β 2	262,144	256
G5-1	G	OUT:H6	Type A	28	+	−	+	+	+	+	−	a1	ξ/β 2	262,144	64

*astA, heat-stable toxin 1; *lpfA*O26, major subunit of long polar fimbriae of STEC O26; *sfpA*, major fimbrial subunit of Sfp fimbriae; *irp*_2_, iron-repressible protein 2; *fyuA*, ferric yersiniabactin uptake receptor; *cdt*, cytolethal distending toxin; EHEC-*hlyA*, enterohemorrhagic *E. coli* hemolysin: *bfpA*, major pilin structural unit bundling; *iha*, iron-regulated gene A homolog adhesin; *espP*, extracellular serine protease; *efa1*, enterohemorrhagic *E. coli* factor for adherence (Efa-1); *pagC*, outer membrane invasion protein.

† MMC, mitomycin C.

‡ Type A is as same as the sequence of O128:H2 isolates (AJ270998 and AJ 010730) in Europe, compared across 1,230 bp; Type B is as same as the sequence of an O63 isolate (AB232172) from Nagasaki, Japan, compared across 1,230 bp.

§ +, positive; −, negative.

‖ OUT, untypeable in O serogroup.

**Figure 3:**
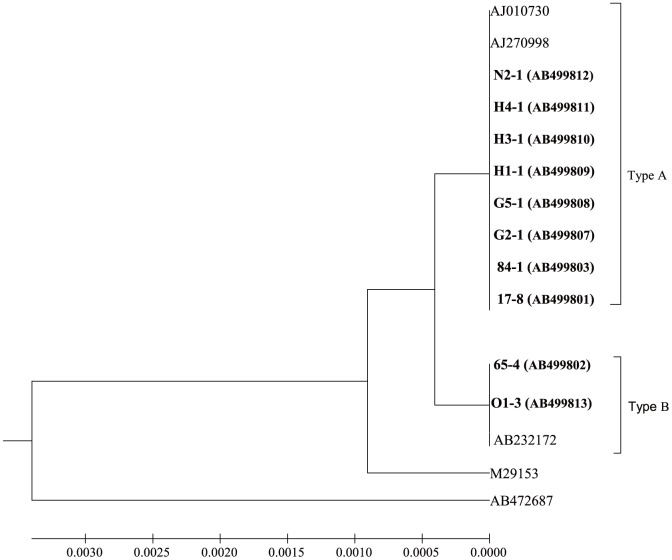
Phylogenetic tree showing *stx*_2f_ nucleotide sequence clusters in this study and *stx*_2f_ sequences in other *Escherichia coli*. The *stx*_2f_ sequence in this study belonged to the branch of *stx*_2f_ genogroups, based on approximately 1.23 kb of sequence from the start codon of subunit A to the stop codon of subunit B. Accession numbers for reference sequences are in parentheses. Nucleotide sequence clusters identified in the current study are indicated in bold. The scale bar indicates the number of nucleotide substitutions per site. Cluster analysis was performed using an unweighted pair-group method with arithmetic average using MEGA 4 software [18]. “Type A” and “Type B” are described in footnote “‡” of Table 2.

**Figure 4:**
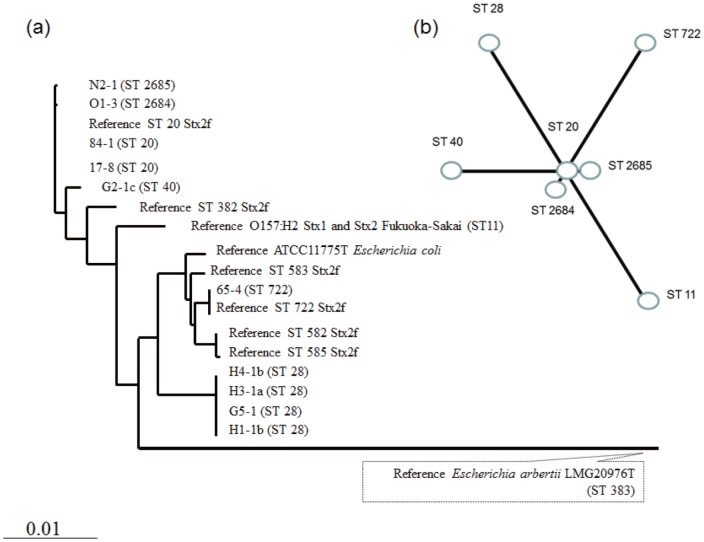
Results of multi-locus sequence typing of Shiga toxin 2f-producing *Escherichia coli* isolates. (a) Phylogenetic tree showing nucleotide sequence clusters of selected isolates with multi-locus sequence typing. Allele sequences for each strain were concatenated in the order *adk*–*fumC*–*gyrB*–*icd*–*mdh*–*purA*–*recA* for a final composite length of 3,423 bp. Reference sequence types (ST) 20, ST 382, ST 583, ST 722, ST 582, and ST 585 are available from the study by Prager *et al*. [1]. Other reference sequences were tested in the present study. The scale bar indicates the number of nucleotide substitutions per site. *Escherichia coli* type strain ATCC11775^T^ and *Escherichia albertii* type strain LMG20976^T^ are included as reference. (b) A minimum spanning tree was also constructed using Prim's algorithm from the PubMLST site (http://pubmlst.org/, accessed May 2, 2013).

The isolates contained several virulence factors, as well as two different *stx*_2f_ STs (Table 2). Eight out of 10 isolates had *stx*_2f_ STs that were previously identified in European isolates [2], whereas two other isolates had sequences that showed identity to a human isolate from Nagasaki, Kyushu [27] (Fig. 3). The major characteristics of the virulence factors are summarized in Table 2. The gene that was detected by PCR with the primers for *sfpA* was confirmed as *sfpA* by DNA sequencing. The sequences from five isolates were completely identical to each other (525/525 bp, 100%), and only differed at 10 bases (10/525) from the *sfpA* sequence reported for *E. coil* O157:H- (AF401292). All of the isolates harbored *cdt* (we did not perform sub-typing) and *astA*, as well as *eae*. The dominant intimin type was type β in the 3′ terminal region, and b1 type in the 5′ terminal region. The MLST results revealed six STs in the selected isolates (Table 2, Fig. 4). No correlation was observed among *stx*_2f_-sequence patterns, *eae*-types, and STs, although presence-absence patterns of virulence related genes might be correlated with intra-isolate STs (Table 2).

### Database accession numbers

The DDBJ accession numbers for the stx2f sequences of the isolates are AB499801–AB499803 and AB499807–AB499813. The *sfpA* sequences are submitted under numbers AB826561–AB826565. The sequences generated by multi-locus sequence typing (MLST) are available from the MLST database (http://mlst.ucc.ie/).

## Discussion

The current study produced three important findings in regard to the isolation and characteristics of STEC2f in pigeons in Japan. First, STEC2f was isolated from pigeon excrement in Kyushu, Japan. The percentage of *stx*_2f_-positive droppings varied from 0–17% across the 14 locations examined. Second, some of the *stx*_2f_-gene fragment sequences identified in the current study were previously identified in Europe [2], and STEC2f O145:H34, ST 722, which has been isolated in Europe [1], was also obtained from pigeon droppings at location I in the present study. Last, the isolates varied in their serotypes, phenotypes, and genotypes. In particular, some isolates contained *sfpA*, which is reportedly restricted to some STEC strains [28], suggesting horizontal transfer of this virulence gene.

The isolation of STEC2f from pigeon droppings in Kyushu adds to the global surveillance data on STEC2f from pigeons. STEC2f from pigeons has previously been reported in other countries, including Finland [8], Germany [6], Italy [9], and India [29]. Kobayashi *et al* [7] reported that the frequency of STEC2f carriage in pigeons in Tokyo Bay, Japan, was 3.0%, while 3.5% of 29 studied pigeons in Finland carried STEC2f [8] Groβmann *et al*. [6] reported that 30.0% and 37.0% of 366 German pigeon dropping samples contained STEC2f, as analyzed by PCR and DNA-DNA hybridization, respectively. However, as not all of our samples came from separate pigeons, comparing our results with previous studies is difficult. Further investigation with direct sampling from pigeon cloaca is required to measure carriage rates of STEC2f in pigeons in Kyushu, Japan. The present STEC2f-positive isolates showed various lineages and harbored several virulence factors. All of the selected isolates contained *eae*, *astA*, and *cdt*, while some of these isolates also had “the high virulence pathogenicity island” characterized by the genes *irp*_2_ and *fyuA*, in addition to genes for the major fimbrial subunit, LPFO26 (*lpfA*_O26_) [5]. Moreover, one O119:H21 and four O-untypeable:H16 isolates harbored *sfpA* (Table 2), which encodes the Sfp protein, the major pilin subunit of STEC O165:H2 and sorbitol-fermenting STEC O157:NM strains [28]. This indicates the horizontal transfer of this virulence gene to other serotypes, which is a novel and an important finding. The presence of *eae*, the locus coding for enterocyte effacement, in the STEC2f-positive strains is similar to previous findings [6], [9]. In particular, the confirmation of the β *eae* subtype in some of the present isolates is consistent with reports by Morabito *et al.* [9]. In other bacteria, some *E. albertii* strains examined in a report by Ooka *et al*. [30] encoded both *eae* (subtype N1.1 or N2) and *stx*_2f_. In the present study, tested strains were confirmed to be *E. coli*, not *E. albertii*, using specific PCR and MLST analyses (Fig. 4) used previously by Ooka *et al*. to identify *E. albertii* [30]. In the current study, none of the isolates contained *efa1*, which is expressed following expression of *eae* and plays an important role in intestinal colonization by STEC strains [31]. Sonntag *et al*. reported the presence of *lpfA*_O26_ but not *iha*, *efa1*, *sfpA*, *espP*, *pagC*, *irp*_2_, *fyuA*, or *cdt* in their STEC2f-isolates, while our results showed the presence of *sfpA*, *irp*_2_, *fyuA*, and *cdt*, as well as *lpfA*_O26_, in some isolates. Further studies are needed to evaluate potential pathogenicities of the STEC2f strains. Six STs were identified in this study. ST 28, ST 2684, and ST 2685 are newly documented STs for STEC2f. These results suggest that several lineages of STEC2f are present in Kyushu, Japan, some of which may be hazardous to humans. The discrepancies among *stx*_2f_-sequences, *eae*-types, and presence-absence patterns of virulence-related genes in each of the STEC2f lineages, as defined by MLST, suggest that further studies are needed to determine how and when each *E*. *coli* lineage acquired Shiga toxin phage, *eae*, or virulence-related genes.

Serotyping and MLST revealed that there may be a relationship between Japanese and European STEC2f strains. This is the first study to document serotype O119:H21 as being STEC2f, while O128:HNM, O145:H34, and O152:HNM were previously reported as human or pigeon STEC2f isolates [1], [3]–[5], [27], [29], [32], [33]. In particular, the O128 group, including O128:HNM, have been isolated by many laboratories in Japan [4] and Europe [1], [5], [32]. In addition, the 1,230-bp *stx*_2f_ sequence of some of the present isolates was the same as that identified for European isolates. STEC2f O145:H34, which is ST 722, was detected in both German human samples [1] and in Japanese pigeon excrement. These results suggest that STEC2f strains in Kyushu, Japan, and those in Europe may be in the same clonal complexes or lineage. It is important to identify the source of transmission of this pathogen, harboring several virulence-related genes, between Japan and Europe. Our findings suggest that STEC2f strains in pigeons represent a potential hazard in Japan. The findings contribute to public health in Kyushu and other parts of Japan, where STEC2f, and its relation to pigeons has not been reported. Because commercial *stx* PCR primers seldom detect the *stx*_2f_ gene [17], we recommend testing *E. coli* isolates with *stx*_2f_-specific PCR primers. This knowledge is particularly useful for public health in Japan.
